# Right Atrial Dimension Is Related to Bidirectional Block Parameters After Cavotricuspid Isthmus Ablation in Patients with Typical Atrial Flutter

**DOI:** 10.3390/jcm15114008

**Published:** 2026-05-22

**Authors:** Mustafa Lutfullah Ardıc, Emre Sezici, Hilmi Erdem Sumbul, Fadime Koca, Mevlut Koc

**Affiliations:** 1 Department of Cardiology, University of Health Sciences, Adana Health Practice and Research Center, 01230 Adana, Turkey; drmustafaardic@gmail.com (M.L.A.); emresezici@gmail.com (E.S.); 2 Department of Internal Medicine, University of Health Sciences, Adana Health Practice and Research Center, 01230 Adana, Turkey; erdemsumbul@gmail.com; 3 Department of Cardiology, Cukurova State Hospital, 01170 Adana, Turkey; drfadimekoca@gmail.com

**Keywords:** atrial flutter, radiofrequency ablation, bidirectional conduction block, right atrial dimension

## Abstract

**Background:** There is insufficient data in the literature regarding the relationship between bidirectional conduction block (BCB) parameters and right atrial dimension (RAd). In our study, we aimed to evaluate the relationship between RAd and BCB parameters in typical atrial flutter (AFL) patients. **Methods:** This prospective study included 197 patients who underwent successful RFA with a diagnosis of typical AFL between 2020 and 2025. We performed RAd and BCB measurements in addition to routine assessments in all patients. The presence of BCB was confirmed by measurement of the CTI double-potential peak-to-peak interval (DPPPI) during CSp pacing, as well as by the difference between the conduction times from isthmus lateral-to-medial pacing to the CSp stimulus (ΔI-CS = LI-to-CSp − MI-to-CSp) and the difference between the conduction times from CSp pacing to the lateral and medial isthmus sites (ΔCS-I = CSp-to-LI − CSp-to-MI). Patients were divided into 3 groups according to RAd: Group-I: <2.2 cm/m^2^, Group-II: 2.2–2.4 cm/m^2^, and Group-III: >2.4 cm/m^2^. **Results:** The number of patients in Groups I, II, and III was determined to be 99, 55, and 43, respectively. RA-volume, LVEF, RAd, and LAd values, as well as LI-to-CSp, ΔI-CS, CSp-to-LI, ΔCS-I, and DPPPI durations, significantly increased from Group I to Group III. MI-to-CSp and CSp-to-MI durations also increased from Group I to Group III; being Group I was significantly lower than Groups II and III. When DPPPI durations were categorized as ≤110 ms, 110–119 ms, 120–129 ms, and ≥130 ms, a significant difference was observed among the groups, and all Group II–III cases were found to have a DPPPI duration of ≥120 ms. In Groups II and III, the proportions of patients with DPPPI ≥ 130 ms were 46% and 84%, respectively. **Conclusions:** Our study showed that cavotricuspid-isthmus BCB parameters, which are indicators of successful AFL ablation, are closely and significantly related to RAd.

## 1. Introduction

Typical atrial flutter (AFL) is a cavotricuspid isthmus (CTI)-dependent macro-re-entrant atrial tachycardia associated with an increased incidence of stroke, heart failure, atrial fibrillation (AF), and death [1,2,3,4]. AFL is one of the most common atrial tachyarrhythmias, with an incidence increasing with age, ranging from 88 to 317/100,000 [1,2,5].

Typical AFL is often classified as counter-clockwise and clockwise according to the direction of activation [2]. In typical AFL, atrial activation descends from the right atrium (RA) free wall, slows down as it passes through the CTI region, then ascends through the RA septum, and then passes through the superior anterior-posterior vena cava before returning to the RA free wall. This type of activation is called counter-clockwise AFL [2]. Activation of the same anatomical regions in the opposite direction is called clockwise AFL. In both cases, the left atrium (LA) is depolarized passively [2].

Radiofrequency ablation (RFA) treatment is generally recommended for AFL patients who do not respond to medical treatment [1,2]. The target of ablation treatment is the CTI region. The return to sinus rhythm is not considered as a sign of successful ablation, but rather the development of a permanent and effective bidirectional conduction block (BCB) by creating an effective lesion set in the CTI region [1,2,6,7,8,9,10,11]. Although different methods are used to detect the presence of BCB, the most preferred methods in the clinic are demonstrating the absence of conduction in the CTI lesion region from measurements obtained by differential pacing (medial and lateral to the ablation line) [6,7,11], similarly measuring transisthmus conduction intervals with pacing methods [9], measuring two double potential peak-to-peak intervals obtained in the CTI region (DPPPI > 110 ms) [8,10] and finally investigating the presence of residual conduction gap with local electrogram mapping in the ablation line using 3D methods [10].

In AFL cases, as in AF, there is a direct increase in RA dimension (RAd) due to arrhythmia and arrhythmia-related factors, and the increase in RAd regresses after RFA [10,12,13]. To our knowledge, there are no data in the literature regarding the relationship between the parameters that are determinants of BCB after the CTI line and RAd. We hypothesized that in cases with RA dilation, differential pacing parameters, transisthmus conduction intervals, and DPPPI durations may be prolonged as a result of the prolongation of both counter-clockwise and clockwise conduction pathways. Our study aimed to evaluate whether there is a relationship between BCB parameters and RAd obtained after CTI ablation performed in typical AFL cases.

## 2. Methods

### 2.1. Study Population

Patients who underwent electrophysiological study (EPS) and 3D RF ablation with symptomatic and persistent AFL in our hospital’s arrhythmia clinic between 2020 and 2025 were enrolled in this prospective study. Patients with CTI-dependent AFL were identified in the EPS. A power analysis (80% power and *p* < 0.05) was performed to determine the number of patients to be included in the study, considering previous studies and their results. This analysis indicated that at least 100 patients were sufficient for inclusion. After exclusion of patients who have an exclusion criteria, 197 patients who underwent ablation for CTI-dependent AFL were included in the study. Patients ≤18 years of age, documented AF, AFL cases other than typical AFL, congenital heart disease, those who have undergone cardiac surgery, those with a history of ablation, those receiving active antiarrhythmic therapy during RF ablation, pregnant women and those less than 3 months postpartum, those with chronic inflammatory disease, those undergoing dialysis, those with known end-stage renal failure, moderate to severe valvular heart disease, known systolic and diastolic HF, hematological disease, active thyroid disease, chronic liver disease, and active cancer were excluded from the study. The necessary permissions for the study were obtained from the ethics committee of our hospital (Date and number: 8 April 2020 and 54-795). All patients included in the study were informed about the study and signed an informed consent form.

After patients were included in the study, their medical histories and physical examinations were performed. Demographic parameters such as age, gender, hypertension, diabetes mellitus, hyperlipidemia (HPL), history of previous cerebrovascular events (CVE), and vascular disease were recorded. Resting heart rate was measured. The active medical treatments received by the patients were recorded. Weight and height were measured, and body mass index and body surface area were determined.

Routine laboratory tests were performed for all cases at the time of admission. Complete blood count, plasma glucose, blood urea nitrogen, creatinine, estimated glomerular filtration rate (eGFR), sodium, potassium, total cholesterol, low-density lipoprotein cholesterol, high-density lipoprotein cholesterol and triglycerides, and high-sensitivity C-reactive protein levels were measured using automated devices (Abbott Aeroset, Roseville, MN, USA) and kits (Abbott). eGFR levels were calculated using the Modification of Diet in Renal Disease (MDRD) formula. Left atrium (LA) end-diastolic diameter, RAd minor axis, and left ventricular ejection fraction (LVEF) were calculated automatically for all cases according to Simpson’s rule [14].

### 2.2. Diagnosis of Atrial Flutter

Patients were evaluated for AFL diagnosis by two electrophysiologists (FK and HK) without being aware of the clinical presentation, risk factors, and the fact that the patient was included in the study. In case of inconsistent results between the two electrophysiologists (interobserver inconsistency), the final decision was made by the third electrophysiology specialist within our clinic (MK). 12-lead electrocardiography was used for AFL diagnosis. The latest current guideline criteria were used for AFL diagnosis [1,2]. Accordingly, typical AFL was defined as: (i) regular atrial activity, (ii) atrial cycle length of 250–330 bpm, (iii) regular and variable atrial conduction in the R-R interval (1:1–2:1–3:1 etc.), (iv) negative sawtooth pattern of P waves in inferior leads, (v) positive P wave in V1 lead for counter-clockwise AFL and (vi) positive and broad P waves in inferior leads, (vii) frequent bimodal negative P wave in V1 lead for clockwise AFL.

### 2.3. Electrophysiologic Study, Mapping and Radiofrequency Ablation Protocol for Atrial Flutter

All patients underwent EPS after at least 5 half-lives of antiarrhythmic drugs, and exclusion of atrial thrombus by transesophageal echocardiography. AFL ablation was performed under sedoanalgesia with fentanyl-midazolam combination via the supervision of an anesthesiologist. EPS was performed using a WorkMate Claris™ device (St. Jude Medical, St. Paul, MN, USA). The right and left inguinal regions were prepared for the EPS procedure. Two diagnostic catheters, one quadripolar and one decapolar, were placed in the high right atrium and the other in the coronary sinus, respectively, via the left femoral vein. A steerable sheath (Agilis NxT 8.5 F, St. Jude Medical, St. Paul, MN, USA) was placed via the right femoral vein. If a prominent p wave was observed on surface ECG, ‘windows of interest’ were determined according to the ‘De Ponti’ formula, and if no p wave was observed, ‘windows of interest’ were determined by calculating 90% of the tachycardia cycle [15]. First, anatomical and electrical mapping of the RA and, if necessary, the LA via septostomy was performed using a high-resolution mapping catheter (PentaRay NAV, Biosense Webster, Diamond Bar, CA, USA). AFL was mapped using 3D mapping systems (CARTO 3, Biosense Webster, Diamond Bar, CA, USA). In RA mapping and ablation procedures, all patients were administered 50–100 IU/kg heparin according to their weight, and the procedure was continued while maintaining an activated coagulation time of 300–500 s.

For AFL ablation, a Thermocool Smarttouch SF (Biosense Webster, Diamond Bar, CA, USA) irrigated catheter was used. At this stage, a transmural continuous linear lesion was created between the tricuspid annulus and the inferior vena cava. Ablation was applied to these areas with irrigated RFA catheters at 35 Watts with a contact force ranging from 5–30 g depending on the characteristics of the target tissue and the presence of surrounding cardiac structures, for 30 s at each point until the local A waves disappeared and with a distance of <4 mm between the two lesions (point-by-point) [16]. Following ablation, after a 20–30 min waiting period, patients were given isoproterenol at a dose of 20–30 μg/min to investigate reconnection. After AFL ablation, BCB was assessed in all cases using differential pacing. In addition, a DPPPI duration of ≥110 ms was accepted as the cutoff value for successful CTI block in all patients. The CTI line was also evaluated using the RF ablation catheter. In selected and equivocal cases, local electrogram analysis and activation mapping were additionally performed. In patients with reconnection, areas with conduction gaps were identified, re-ablation was performed, and bidirectional block was achieved.

### 2.4. Assessment of Cavotricuspid Isthmus Conduction Block

**Figure 1:**
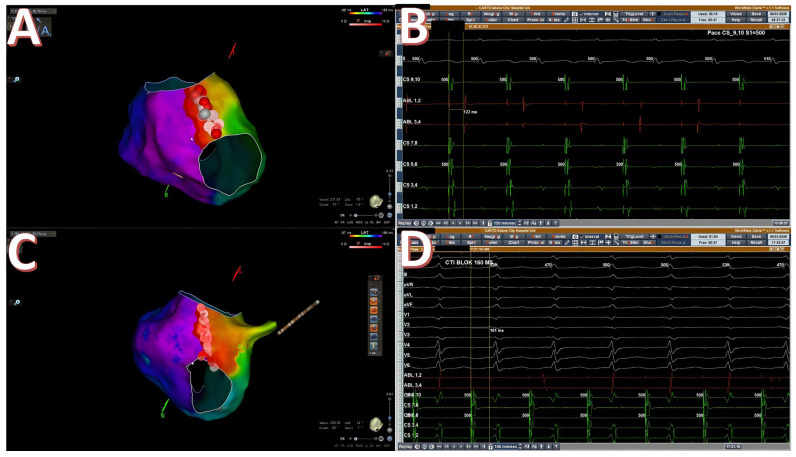
(**A**) A case study of a patient with a minor axis dimension of 2.12 cm/m^2^ in the right atrium and right atrium volume 231 mL with 3D mapping system who underwent radiofrequency ablation (RFA) with 3D mapping: An effective and permanent linear line was created in the cavotricuspid isthmus (CTI) region after RFA. (**B**) Then, bidirectional conduction block was determined by pacing from CSp and conduction interval was evaluated from CSp to lateral isthmus time (122 ms). (**C**) A case study of a patient with a minor axis dimension of 2.42 cm/m^2^ in the right atrium and right atrium volume 356 mL with 3D mapping system who underwent RFA with 3D mapping: An effective and permanent linear line was created in the CTI region after RFA. (**D**) Then, bidirectional conduction block was determined by pacing from CSp and conduction interval was evaluated from CSp to lateral isthmus time (160 ms).

**Figure 2:**
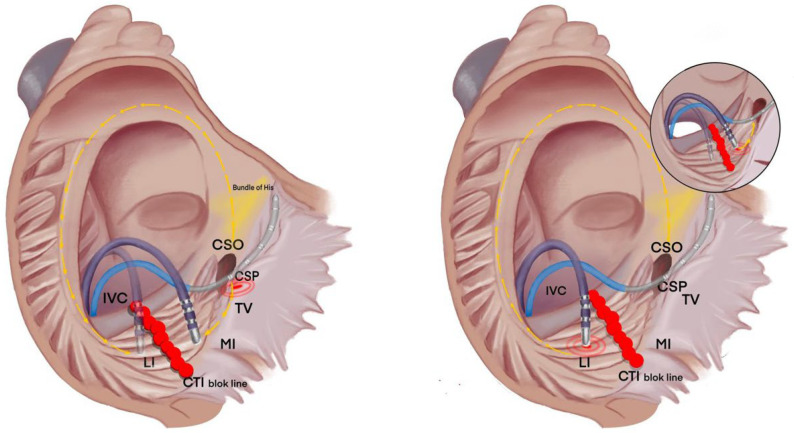
The anatomical image of the right atrium (RA) schematically illustrates the differences in bidirectional conduction block measurements obtained from differential pacing assessment after typical atrial flutter ablation, according to three different minor axis dimension values of the RA.

Durable and complete BCB in CTI was accepted as the end-point of the procedure. The first method for the presence of BCB was accepted as DPPPI > 110 ms on the ablation line (the region from the tricuspid annulus to the inferior vena cava) with proximal coronary sinus (CSp) pacing. As a second method, the difference between the times of arrival of stimulation to CSp [LI-to-CS and MI-to-CS] from 10 mm lateral (lateral isthmus—low lateral right atrium) and 10 mm medial (medial isthmus) of the ablation line and the time of arrival of the stimulation to the lateral and medial isthmus [CS-to-LI and CS-to-MI] from CSp pacing were used [8]. Third and finally, transisthmus conduction interval measurements were performed using the obtained parameters; ΔI-CS (LI-to-CSp time − MI-to-CSp time) and ΔCS-I (CSp-to-LI time − CSp-to-MI time) were performed (Figure 1 and Figure 2).

### 2.5. Statistical Analysis

All statistical analyses were performed using IBM SPSS Statistics version 25.0 (SPSS Inc., Chicago, IL, USA). Variables were categorized as either categorical or continuous. Categorical variables were presented as frequencies and percentages, while continuous variables were expressed as mean ± standard deviation (SD). The normality of distribution for continuous variables was assessed using the Shapiro–Wilk test and, additionally, the Kolmogorov–Smirnov test. Comparisons of continuous variables across three groups were performed using one-way ANOVA for normally distributed data or the Kruskal–Wallis one-way ANOVA for non-normally distributed data. For multiple comparisons of normally distributed variables, Scheffé or Games–Howell post-hoc tests were applied, depending on the homogeneity of variances. For non-normally distributed variables, Bonferroni-adjusted Mann–Whitney U tests were used for pairwise comparisons. Fisher’s exact test was applied to evaluate differences in non-numeric categorical parameters. The parameters associated with BCB measurements were identified using univariate correlation analysis with Pearson’s and Spearman’s correlation methods. Statistically significant parameters were included in linear regression analysis, and the parameters most closely related to BCB measurements were determined. For all multivariable evaluations (linear regression analyses), univariate comparisons were initially performed. Subsequently, variables with a *p*-value < 0.25 in the univariate analysis were entered into the multivariable model using a stepwise forward-selection procedure. A *p*-value < 0.05 was considered statistically significant.

## 3. Results

Among 242 AFL patients who presented to our clinic with a diagnosis of AFL, AFL ablation was performed in 218 of these patients after careful evaluation. After patients meeting the exclusion criteria were excluded, 197 patients (129 men, 68 women, age 61.8 ± 12.1 years) with CTI-dependent AFL who underwent successful 3D RF ablation were included in the study. The procedure was unsuccessful in 3 patients (1.5%) with typical AFL, and these cases were not included in the study. Complications developed in only 2 patients during the RFA procedure (1 patient with mild pericardial effusion that responded to medical treatment and 1 patient with non-invasive A-V fistula formation in the left femoral region). According to the latest ASE and EACI guideline recommendations, minor axis RA > 2.2 cm/m^2^ was considered RA dilation. Patients included in the study were divided into three groups based on their RAd values: normal (<2.2 cm/m^2^), mild-moderate dilation (2.2–2.4 cm/m^2^), and severely dilated (>2.4 cm/m^2^) (Group I, Group II, and Group III, respectively). The number of patients in Groups I, II, and III was determined to be 99, 55, and 43, respectively. All parameters in the study were compared between the groups. In addition, parameters related to BCB measurements after ablation were evaluated.

### 3.1. Demographic, Clinical, and Medical Treatment Data of the Study Groups

The demographic, clinical, and medical treatment data of the study groups are shown in Table 1. In Group II and III, female gender, HPL, and CVE frequency were found to be significantly higher compared to Group I. Other demographic, clinical, and medical treatment data were similar across all groups.

### 3.2. Laboratory and Echocardiography Data of the Study Groups

The laboratory and echocardiography data of the study groups are shown in Table 2. It was determined that LVEF decreased and LAd increased from Group I to Group III and were significantly different among all groups (Table 2). Other laboratory and echocardiography data were similar among the groups.

### 3.3. Electrophysiologic Data

The mean LI-to-CSp duration was shown to be 148 ± 7.7 (range 123–163), the MI-to-CSp duration was 23.6 ± 2.5 (range 18–28), the CSp-to-LI duration was 142 ± 7.4 (range 118–157), the CSp-to-MI duration was 21.8 ± 2.5 (range 16–27), and the DPPPI duration was 127 ± 7.4 (range 103–142). The electrophysiology study and ablation data of the study groups are shown in Table 3. It was determined that RA volume, LI-to-CSp, ΔI-CS, CSp-to-LI, ΔCS-I, and DPPPI durations increased from Group I to Group III, and there was a statistically significant difference between all groups. It was found that MI-to-CSp and CSp-to-MI durations increased from Group I to Group III; Group I being significantly lower than Groups II and III; and similar between Groups II and III. Significant differences were found between groups when DPPPI durations were grouped as ≤110 ms, 110–119 ms, 120–129 ms, and ≥130 ms (*p* < 0.001). All cases with RA dilation (Group II and Group III) had a DPPPI duration of ≥120 ms. Furthermore, the frequency of patients with a DPPPI duration of ≥130 ms was 25 (46%) and 36 (84%) in Groups II and III, respectively. Other electrophysiology studies and ablation data were similar between the groups.

### 3.4. The Parameters Associated with Bidirectional Block Measurements After Successful Cavotricuspid Isthmus Ablation

**Figure 3:**
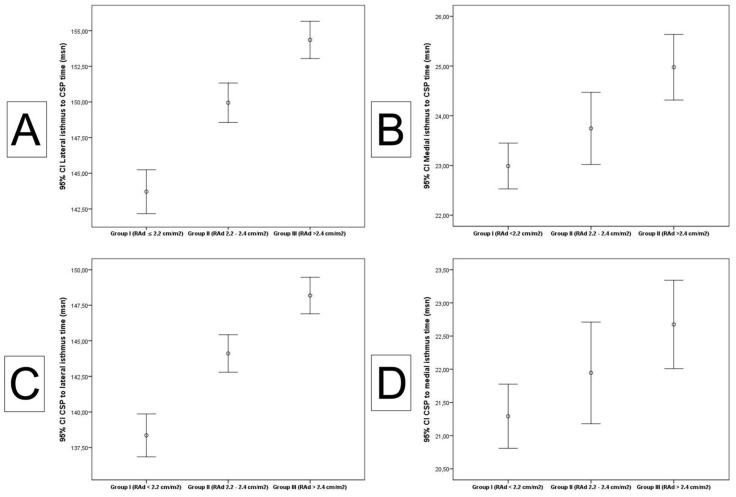
Error bar graph showing the variation of bidirectional conduction block parameters after atrial flutter ablation according to right atrium minor axis dimension groups. (**A**) Lateral isthmus to coronary sinus proximal (CSP) time; (**B**) Medial isthmus to CSP time; (**C**) CSP to lateral isthmus time; (**D**) CSP to medial isthmus time.

**Figure 4:**
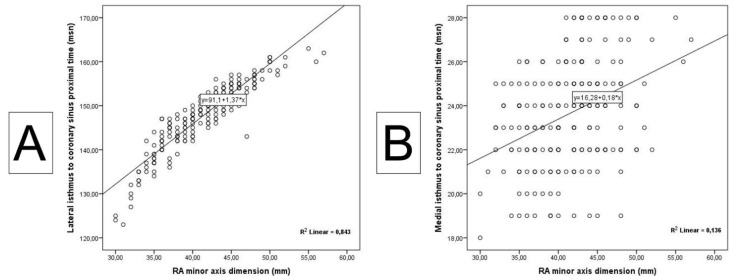

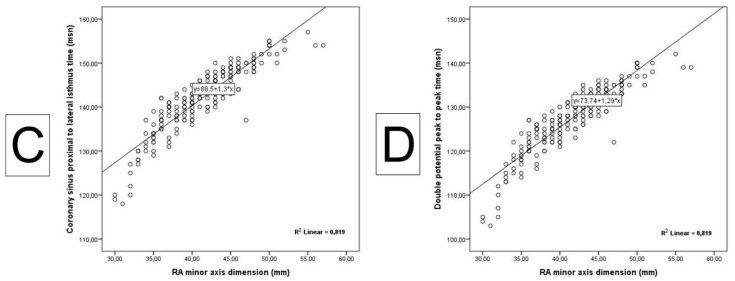
Scatter/Dot plots showing the relationship between bidirectional conduction block parameters and right atrial minor axis dimension after atrial flutter ablation. (**A**) Lateral isthmus to coronary sinus proximal time; (**B**) Medial isthmus to coronary sinus proxi-mal time; (**C**) Coronary sinus proximal to lateral isthmus time; (**D**) Double potential peak-to-peak time.

Demographic, clinical, laboratory, and echocardiography parameters that were significantly associated with BCB measurements in the univariate analysis are summarized in Table 4. Linear regression analysis was performed with these parameters (Table 5). A very close relationship was found between the RA minor axis and the 3D calculated RA volume values, with the DPPPI, LI-to-CSp, MI-to-CSp, CSp-to-LI, and CSp-to-MI durations, with the RA minor axis being more prominent. Furthermore, a close relationship was determined between LAd and the LI-to-CSp, CSp-to-LI, and DPPPI durations (Table 5). For the regression model, multicollinearity was assessed using variance inflation factor (VIF) values. All VIF values were below 2, indicating a low degree of multicollinearity. The relationship between the RA minor axis and the DPPPI, LI-to-CSP, MI-to-CSp, and CSp-to-LI is shown in Figure 3 and Figure 4.

$$\begin{array}{l} R_{Adjusted}^{2}\\ =Lateral\;to\;CSO\;\left(0.873\right),\;septal\;to\;CSO\;\left(0.205\right),\;CSO\;to\;lateral\;\left(0.851\right),\;CSO\;to\;septal\;\left(0.121\right)\;and\;DP\;peak\;to\;peak\;(0.848) \end{array}$$

in multivariate analyses.

## 4. Discussion

This study has several important findings, which can be summarized as follows: (1) Approximately 50% of cases diagnosed with typical AFL and undergoing RFA have RA dilation (mild/moderate 28% and severe 22%), (2) In cases with RA dilation, there are higher BCB-related parameters compared to those without RA dilation, (3) This increase in the duration of BCB-related parameters is even greater in cases with severe RA dilation than in those with mild/moderate dilation, (4) In cases without RA dilation, consistent with the literature, it was demonstrated that the DPPPI duration was ≥110 ms in the vast majority (94%) of patients with typical AFL., (5) In cases with RA dilation, all patients who underwent successful typical AFL ablation were found to have a DPPPI duration of ≥120 ms.

RFA treatment is recommended for all symptomatic and persistent cases of AFL because medical treatment is insufficient [2]. In a meta-analysis of 18 studies and 1323 patients, the success rate of RFA in AFL cases was reported as 91.7%, and the risk of complications was 0.5% [17]. In our clinic, RFA has been applied to typical AFL patients for approximately 16 years, initially with a fluoroscopy approach, and for the last 10 years, routine procedures have been performed using a 3D mapping system. All our data were obtained using a 3D mapping system. In our study, the success rate of AFL ablation was shown to be 98.5%, and the frequency of complications was 1%. After AFL ablation, patients have a risk of stroke due to AFL or AF occurrence, and anticoagulant treatment is recommended in accordance with the risk factors [1]. In our study, it was determined that AFL patients received appropriate anticoagulant treatment before the procedure. In our study, 4 patients had a history of stroke, and all 4 patients had dilated RA.

In typical AFL cases, the aim of RFA treatment is to restore sinus rhythm in patients and to create complete and permanent BCB along the CTI line. Testing of BCB within the CTI was first demonstrated by Cauchemez et al. in 1996 [11]. In this study, the activation pattern was examined with pacing from two sides of the CTI lesion, forming the basis of differential pacing. It was shown that pacing from the lateral side of the CTI caused descending activation of RA septum; while pacing from the medial or septal part of the CTI caused descending activation of RA lateral free wall [11]. In later years, Chen et al. suggested a similar method [6]. In the study by Chen et al. [6], similar to the previous study, the presence of BCB was shown by evaluating conduction delays obtained by pacing from four different regions, two from the medial and two from the lateral side of the CTI lesion. In another study, Shah D et al. [7] described the differential pacing method for the presence of persistent conduction in the CTI region in 2000. In 2001, Tada H et al. [8] reported that BCB can be demonstrated by evaluating DP and measuring DP1-2 duration in the CTI ablation line. In this study, it was shown that a DPPPI duration of ≥110 ms can be used as a cut-off for BCB with high sensitivity and specificity. In 2011, Oral H et al. [9] performed transisthmus conduction interval evaluation for BCB assessment [9]. They reported that an increase of ≥50% in transisthmus conduction interval after RFA can be used as an indicator of BCB after CTI ablation [9].

After the use of 3D mapping systems, the most sensitive BCB assessment is performed by searching for residual conducting gaps using local electrogram mapping along the CTI ablation line. Even though it has high sensitivity, this method requires high resolution mapping along the linear lesion line. Therefore, differential pacing methods are preferred. In our clinic, after AFL ablation, we usually perform BCB assessment with differential pacing, mostly using a cut-off value of ≥110 for DPPPI duration, and in some selected cases, with local electrogram and activation mapping.

In AFL cases, as in AF, long-term atrial remodeling, increased volume and pressure load, and accompanying tricuspid regurgitation lead to RA enlargement [10,12,13,18]. This increase in RA dimensions alters electrical conduction times due to increased distance between anatomical structures. Above-mentioned previously conducted studies failed to evaluate the effect of RA dilation on BCB measurements.

The most important difference between our study and previous studies [6,7,8,9,10,11] is that all procedures were performed with 3D mapping, and therefore we were able to be sure that differential pacing, DP1-2 and transisthmus conduction interval measurements were made from fixed anatomical regions. In the study by Chen et al. [6], the LI-to-CSp time was reported as 148.3 ± 24.5 ms (range 105–210). In our study, although more precise measurements were obtained with 3D mapping systems, the LI-to-CSp time was shown to be 148 ± 7.7 ms (range 123–163). As we grouped patients according to RAd; we found LI-to-CSp times to be 144 ± 7.7 ms, 150 ± 5.1 ms, and 154 ± 4.2 ms in patients without RA dilation, with mild/moderate RA dilation, and with advanced RA dilation, respectively. Our study showed that not only the LI-to-CSp time, but also other parameters increased with RA dilation. One of the methods for BCB testing is transisthmus conduction interval assessment. We determined that the ΔCS-I and ΔI-CS times obtained from this assessment were different in cases without RA dilation, with mild/moderate RA dilation, and with advanced RA dilation. We specifically determined that a transisthmus conduction interval ≥110 ms would be useful, particularly in cases with RA dilation. Another BCB parameter is DPPPI duration. In our study, this measurement value, like other BCB parameters, was found to increase in cases with RA dilation. Contrary to the ≥110 ms value in the literature, the DPPPI duration was ≥120 ms in all cases with RA dilation, and even ≥130 ms in 84% of the patients with severely dilated RA. We believe that the increase in BCB measurements observed in our study may be related to prolonged conduction time secondary to RA dilatation. The increased BCB distances associated with increased RAd observed in our study should be validated by further studies with larger patient populations and findings similar to ours. In addition, it should be kept in mind that these measured distances are approximate values and may vary depending on patient-related factors.

Atrial fibrillation (AF) is increasingly recognized not only as an arrhythmia but also as a clinical manifestation of atrial cardiomyopathy (AtCM) [19]. AtCM is a progressive and multifaceted disease involving structural, electrical, mechanical, and molecular remodeling of the atrial myocardium [19]. AtCM often precedes the onset of AF, promotes its maintenance, and contributes to thromboembolic risk independently of rhythm status [19]. Current AF guidelines also indicate that AFL may represent a risk factor for thromboembolism similar to AF. Although perhaps not to the same extent as AF, AFL may also lead to AtCM through similar pathophysiological alterations in both the RA and LA. Although AtCM was not directly evaluated in our study, the increase in BCB parameters associated with increased RAd in patients with AFL may be explained by the presence of AtCM-like changes in these patients.

## 5. Limitations

This study has several important limitations. The study was conducted in a single center with a limited number of patients. Stronger results could have been obtained with a multi-center study and an increased number of patients. We did not include patients who had typical AFL and AF to our study. However, these cases can be seen together in clinical practice. It should be evaluated whether similar findings can be obtained by including this group of patients in future studies. Most advanced EP laboratories use intracardiac echocardiography (ICE) guidance during the procedure [20]. We did not use (ICE) guidance in our study. In a study using ICE, the RA anatomical features could be better evaluated. Another indicator of BCB in CTI line is considered to be an increase of 20 ms in the existing DP with incremental pacing [21]. We did not perform this assessment either. Another limitation of our study is that all included patients underwent successful CTI ablation. More meaningful data might have been obtained if unsuccessful cases had also been included and the threshold BCB parameters according to RAd had been evaluated in those patients as well. In addition, all study data were obtained by our own group, resulting in a lack of external validation. Furthermore, we did not follow patients after CTI ablation for AFL recurrence. Individuals who undergo successful AFL ablation are known to have an increased future risk of AF, with reported AF incidence rates ranging from 18% to 50% [22]. Follow-up data regarding AFL recurrence and AF development could therefore have provided more clinically meaningful insights. It should also be considered that all BCB measurements may be subject to potential variability.

Although our findings demonstrated that increased RAd is electrophysiologically associated with altered RA conduction, we believe that histopathological assessment could provide stronger evidence regarding this relationship. In addition, the cutoff values obtained for the parameters used in BCB assessment in our study require validation by further studies before their routine clinical applicability can be established. Previous studies have reported improvement in cardiac size (particularly RA volume index) and function during follow-up after CTI-dependent AFL ablation [13]. However, in our study, we did not evaluate these parameters or perform comprehensive echocardiographic assessment of other right heart structures beyond the RA. Inclusion of such assessments could have strengthened our study findings.

## 6. Conclusions

Our study showed a close and significant correlation between CTI isthmus BCB parameters and increased RAd. Following successful AFL ablation, DPPPI duration, one of the most important parameters for BCB assessment, was found to be ≥120 ms in all cases with increased RAd, and this duration was predominantly ≥130 ms, particularly in patients with advanced RAd enlargement. However, future multicenter, randomized studies with larger sample size are needed to verify these results.

## Figures and Tables

**Table 1:** Demographic and clinical data of study groups undergoing atrial flutter ablation.

Variables	Group I *n* = 99	Group II *n* = 55	Group III *n* = 43	*p*
Age (year)	61.2 ± 12	64.1 ± 10	60.1 ± 14	0.211
Gender (Female), *n*	25 (25%)	21 (38%)	21 (49%)	**0.002**
Hypertension, *n* (%)	62 (63%)	43 (78%)	22 (51%)	0.471
Diabetes mellitus, *n* (%)	15 (15%)	16 (29%)	9 (21%)	0.170
Cerebrovascular event, *n* (%)	0 (0%)	2 (4%)	2 (5%)	**0.042**
Coronary artery disease, *n* (%)	28 (28%)	21 (38%)	13 (30%)	0.526
Hyperlipidemia, *n* (%)	11 (11%)	15 (27%)	10 (23%)	**0.021**
Body mass index (kg/m^2^)	26.9 ± 2.7	27.1 ± 3.0	26.4 ± 2.87	0.123
Heart rate (beat/min)	143 ± 11	141 ± 12	139 ± 7.2	0.154
Anti-coagulant drug use, *n* (%)	87 (88%)	48 (87%)	37 (86%)	0.897
Anti-arrhythmic drug use, *n* (%)	61 (62%)	34 (62%)	31 (72%)	0.322

The values were shown as mean ± standard deviation or *n* (%), Statistically significant *p* values were shown in bold. Group I: Normal right atrial dimension (<2.2 cm/m^2^), Group II: Mild-moderate dilation right atrial dimension (2.2–2.4 cm/m^2^) and Group III: severely dilated right atrial dimension (>2.4 cm/m^2^).

**Table 2:** Laboratory data of study groups undergoing atrial flutter ablation at the time of admission.

Variables	Group I *n* = 99	Group II *n* = 55	Group III *n* = 43	*p*
White blood cell (10^3^/µL)	8.1 ± 2.3	8.6 ± 2.6	8.4 ± 2.4	0.250
Platelet count (10^3^/µL)	241 ± 76	261 ± 90	259 ± 112	0.215
Hemoglobin (g/dL)	13.5 ± 2.1	13.1 ± 2.0	12.8 ± 2.2	0.161
Fasting plasma glucose (mg/dL)	122 ± 62	137 ± 86	128 ± 57	0.439
Creatinine (mg/dL)	0.88 ± 0.30	0.92 ± 0.27	0.96 ± 0.36	0.177
Blood urea nitrogen (mg/dL)	35.7 ± 19	44.1 ± 29	39.8 ± 21	0.085
eGFR (mL/min/1.73 m^2^)	87.9 ± 23	77.1 ± 28	83.5 ± 22	0.051
Sodium (mmol/L)	138 ± 3.2	138 ± 3.7	138 ± 3.5	0.972
Potassium (mmol/L)	4.50 ± 0.45	4.49 ± 0.56	4.55 ± 0.65	0.770
hs-CRP (mg/dL)	13.8 ± 2.8	14.9 ± 2.5	19.8 ± 5.5	0.729
Total cholesterol (mg/dL)	171 ± 44	176 ± 61	173 ± 46	0.811
HDL cholesterol (mg/dL)	42.1 ± 11	49.1 ± 25	46.4 ± 18	0.081
LDL cholesterol (mg/dL)	115 ± 29	113 ± 47	111 ± 33	0.797
Triglycerides (mg/dL)	142 ± 88	161 ± 95	165 ± 102	0.340
LVEF (%)	54.4 ± 8.6	49.8 ± 9.4	44.3 ± 10	**<0.001**
LA dimension (mm)	39.3 ± 4.3	42.7 ± 4.9	43.9 ± 3.2	**<0.001**

The values were shown as mean ± standard deviation, eGFR: estimated glomerular filtration rate, HDL: High-density lipoprotein, hs-CRP: High sensitive C reactive protein, LA: Left atrium, LDL: Low-density lipoprotein, LVEF: Left ventricular ejection fraction, statistically significant *p* values were shown in bold. Group I: Normal right atrial dimension (<2.2 cm/m^2^), Group II: Mild-moderate dilation right atrial dimension (2.2–2.4 cm/m^2^) and Group III: severely dilated right atrial dimension (>2.4 cm/m^2^).

**Table 3:** Electrophysiological study and ablation data of study groups undergoing atrial flutter ablation.

Variables	Group I *n* = 99	Group II *n* = 55	Group III *n* = 43	*p*
Tachycardia cycle length, (ms)	233 ± 24	234 ± 17	232 ± 19	0.869
Procedural time (minute)	70.8 ± 12	72.4 ± 8.9	72.5 ± 9.6	0.562
Fluoroscopy time (minute)	15.5 ± 2.9	14.4 ± 2.1	14.7 ± 2.8	0.052
Lateral to CSp time (ms)	144 ± 7.7	150 ± 5.1	154 ± 4.2	**<0.001**
Septal to CSp time (ms)	22.9 ± 2.3	23.8 ± 2.7	24.9 ± 2.2	**<0.001**
CSp to lateral time (ms)	138 ± 7.6	144 ± 4.9	148 ± 4.2	**<0.001**
CSp to septal time (ms)	21.3 ± 2.4	21.9 ± 2.8	22.7 ± 2.2	**0.009**
DP peak to peak time (ms)	124 ± 7.5	129 ± 4.9	133 ± 4.2	**<0.001**
DP peak to peak time ≤110 ms, *n* (%)	6 (6%)	0 (0%)	0 (0%)	
DP peak to peak time 110–119 ms, *n* (%)	21 (21%)	0 (0%)	0 (0%)	
DP peak to peak time 120–129 ms, *n* (%)	50 (51%)	30 (55%)	7 (16%)	
DP peak to peak time ≥130 ms, *n* (%)	22 (22%)	25 (46%)	36 (84%)	
3D RA volume (mL)	202 ± 29	228 ± 24	253 ± 25	**<0.001**

The values were shown as mean ± standard deviation, CSp: Proximal coronary sinus, DP: Dual potential, RA: Right atrium Statistically significant *p* values were shown in bold. Group I: Normal right atrial dimension (<2.2 cm/m^2^), Group II: Mild-moderate dilation right atrial dimension (2.2–2.4 cm/m^2^) and Group III: severely dilated right atrial dimension (>2.4 cm/m^2^).

**Table 4:** The parameters associated with critical isthmus bidirectional block.

	Lateral to CSp Time		Septal to CSp Time		CSp to Lateral Time		CSp to Septal Time		DP Peak to Peak Time	
	*p*	r	*p*	r	*p*	r	*p*	r	*p*	r
BUN	0.001	0.228	0.750	0.023	0.001	0.226	0.949	0.006	0.002	0.221
Creatinine	0.014	0.176	0.500	0.048	0.016	0.172	0.298	0.074	0.015	0.173
eGFR	0.036	−0.150	0.922	−0.007	0.043	−0.145	0.777	−0.021	0.041	−0.147
LVEF	<0.001	−0.579	0.002	−0.219	<0.001	−0.587	0.315	−0.072	<0.001	−0.583
LAd	<0.001	0.685	<0.001	0.252	<0.001	0.681	0.063	0.133	<0.001	0.676
RAd	<0.001	0.918	<0.001	0.369	<0.001	0.905	0.004	0.207	<0.001	0.905
RA volume	<0.001	0.914	<0.001	0.400	<0.001	0.903	0.001	0.229	<0.001	0.904

BUN: Blood Urea Nitrogen, CSp: Proximal coronary sinus, DP: Dual potential, eGFR: estimated glomerular filtration rate, LAd: Left atrium dimension, LVEF: Left ventricular ejection fraction, RAd: Right atrium dimension.

**Table 5:** Linear regression analysis for parameters significantly correlated with critical isthmus bidirectional block.

	Lateral to CSp Time		Septal to CSp Time		CSp to Lateral Time		CSp to Septal Time		DP Peak to Peak Time	
	*p*	β	*p*	β	*p*	β	*p*	β	*p*	β
BUN	0.102	0.061	0.529	−0.058	0.101	0.066	0.258	0.110	0.153	0.058
Creatinine	0.977	0.002	0.154	0.208	0.895	0.008	0.031	0.333	0.876	0.010
eGFR	0.441	0.042	0.134	0.204	0.498	0.040	0.127	0.218	0.591	0.032
LVEF	0.375	0.031	0.623	0.043	0.943	0.003	0.208	0.116	0.832	0.008
LAd	<0.001	0.157	0.911	0.010	<0.001	0.166	0.788	0.024	<0.001	0.152
RAd	<0.001	0.598	0.031	0.288	<0.001	0.520	0.044	0.245	<0.001	0.478
RA volume	0.048	0.301	0.007	0.946	0.049	0.292	0.034	0.312	0.023	0.353

BUN: Blood Urea Nitrogen, CSp: Proximal coronary sinus ostium, DP: Dual potential, eGFR: estimated glomerular filtration rate, LAd: Left atrium dimension, LVEF: Left ventricular ejection fraction, RAd: Right atrium dimension.

## Data Availability

The datasets generated during and/or analyzed during the current study are available from the corresponding author on reasonable request.
